# Supplementary material for: Leisure‐Time Physical Activity, Sedentary Behavior, and Biological Aging: Evidence From Genetic Correlation and Mendelian Randomization Analyses
Source: Scand J Med Sci Sports. 2025 Jan 10;35(1):e70014. doi: 10.1111/sms.70014 (PMC11723829; doi:10.1111/sms.70014)
Supplement: Supplementary file 2 — Data S2. [file SMS-35-e70014-s003.docx]

**Supplementary File 2. Details of tissue-partitioned Mendelian randomization analysis**

**Tissue-specific gene expression data**

We obtained the eQTL (expression quantitative trait loci) data for brain from a meta-analysis study (N = 1,194, all of European ancestry) of 10 brain regions (anterior cingulate cortex, caudate basal ganglia, cerebellar hemisphere, cerebellum, cortex, frontal cortex BA9, hippocampus, hypothalamus, nucleus accumbens basal ganglia, and putamen basal ganglia)^1^. We obtained the eQTL data of skeletal muscle from GTEx consortium v8 (N = 706, ~80% European ancestry)^2^. The datasets were both downloaded from the SMR (summary Mendelian randomization) website (https://cnsgenomics.com/software/smr/), which has already mapped the eQTL data to the hg19 genome build using the GRCh37 reference assembly^3^.

**Genetic colocalization**

We conducted genetic colocalization using the Bayesian method “*coloc*” to assess whether the same variant is causal in both a GWAS and an eQTL study^4 5^. Colocalization analyses were performed at each locus, encompassing all SNPs located within a 200 kb window around each independent MVPA/LST-associated SNP (*P* < 5×10^-8^ and an LD window of ± 1.0 Mb with $r^{2}$ < 0.001). The colocalization analysis was first conducted with eQTL data derived from brain tissue, and then followed by a separate round with eQTL data from skeletal muscle tissue. The “*coloc*” method provided the posterior probability of five competing hypotheses: PPH0 (no association with either trait), PPH1 (association with GWAS trait only), PPH2 (association with eQTL only), PPH3 (association with both traits, distinct causal variants), and PPH4 (association with both traits, shared causal variant). In our study, a locus was considered colocalized if PPH4 ≥ 0.8, indicating both GWAS and eQTL data are associated with a common causal variant^4^. Based on these results, the initial IVs were divided into two sets of instruments, with each set specifically proxying for “brain-tissue instrumented MVPA/LST” and the “skeletal muscle-tissue instrumented MVPA/LST”. To mitigate the potential impact of strong regional linkage disequilibrium (LD) structure, variants within the Major Histocompatibility Complex (MHC) region (chr6: 25M-35M) were excluded in our analysis. To verify the robustness of our results, we also screened an expanded set of IVs using a more relaxed clumping strategy (*P* < 1×10^-5^ and an LD window of ± 1.0 Mb with $r^{2}$ < 0.001) to repeat our analysis.

**Mendelian randomization analysis**

Exposure-outcome pairs were selected for inclusion in tissue-partitioned MR analysis if they demonstrated both robust effects in univariable Mendelian randomization (MR) and directionally consistent effects in multivariable MR. Tissue-partitioned MR was performed in both univariable and multivariable settings. In univariable setting, we first evaluate the unadjusted effects of phenotypic subcomponents of exposures on outcomes, using each set of tissue-partitioned instruments for the exposures. Then, in multivariable setting, we estimate the putatively genetically predicted effects of exposures instrumented by brain and skeletal muscle tissues independently on outcomes. IVs for the phenotypic subcomponents were weighted by their PPH4 values for each tissue type in multivariable settings, and the model was developed to simultaneously incorporate both sets of tissue-partitioned instruments. Given the reduced number of IVs and the resulting decrease in statistical power, we used a conventional significance threshold of *P* < 0.05 in the analysis.

**Supplementary Figure 1. An overview of the tissue-partitioned Mendelian randomization analysis**

Left panel (A) shows the design of a traditional MR analysis, while right panel (B) illustrates the design of a tissue-partitioned MR analysis. In the multivariable setting, the independent effects of phenotypic subcomponents of exposures are estimated by accounting for the effects of other tissues. IVs for the phenotypic subcomponents were weighted by their PPH4 values for each tissue type. LST (leisure screen time), SNP (single nucleotide polymorphisms).

1. Qi T, Wu Y, Zeng J, et al. Identifying gene targets for brain-related traits using transcriptomic and methylomic data from blood. *Nat Commun* 2018;9(1):2282.

2. The GTEx Consortium atlas of genetic regulatory effects across human tissues. *Science* 2020;369(6509):1318-30.

3. Zhu Z, Zhang F, Hu H, et al. Integration of summary data from GWAS and eQTL studies predicts complex trait gene targets. *Nat Genet* 2016;48(5):481-7.

4. Giambartolomei C, Vukcevic D, Schadt EE, et al. Bayesian test for colocalisation between pairs of genetic association studies using summary statistics. *PLoS Genet* 2014;10(5):e1004383.

5. Leyden GM, Greenwood MP, Gaborieau V, et al. Disentangling the aetiological pathways between body mass index and site-specific cancer risk using tissue-partitioned Mendelian randomisation. *Br J Cancer* 2023;128(4):618-25.
